# Predictors of short-term mortality in cardiogenic shock: insights from an Egyptian multicenter registry

**DOI:** 10.1186/s43044-024-00525-y

**Published:** 2024-07-26

**Authors:** Hesham S. Taha, Ahmed Gohar, Walid Ammar, Hossam Alhossary, Ahmed Adel, Reda Diab, Hala Mahfouz, Mirna M. Shaker, Mina Samy

**Affiliations:** 1 Department of Cardiology, Faculty of Medicine, Cairo University, 27 Nafezet Sheem El Shafae St Kasr Al Ainy, Cairo, 11562 Egypt; 2 Shark Al Madina Hospital, Alexandria, Egypt; 3 Menoufia University, Shibin El Kom, Egypt

**Keywords:** Cardiogenic shock, Acute coronary syndrome, Clinical outcome, Predictors of short-term outcome

## Abstract

**Background:**

Cardiogenic shock (CS) remains a major cause of morbidity and mortality, particularly in developing countries where there are limited resources and a lack of data on CS outcomes. This study aimed to investigate 30-day all-cause mortality in Egyptian patients with CS at tertiary referral centers.

**Results:**

This prospective, observational multicenter registry analyzed 16,681 patients from six cardiac centers, to evaluate the incidence, causes and predictors of CS-related mortality. Among the 529 diagnosed CS patients, 68.2% had an ischemic etiology. No discernable variations were observed in clinical or laboratory features, as well as mortality rates, between ischemic and non-ischemic CS patients. Within 30 days, 210 deaths (39.7%) occurred. Non-survivors with ischemic CS had a higher prevalence of diabetes, worsening renal function, and were more likely to receive multiple inotropes. Mortality did not significantly differ between acute coronary syndrome patients with ST elevation myocardial infarction (STEMI) and non-STEMI (NSTEMI) (42.7% vs. 43.7%, *p* < 0.887). However, anterior STEMI patients had significantly higher mortality than those with inferior STEMI (49.5% vs. 21.6%, *p* < 0.003). Multivariate regression analysis identified predictors of mortality in CS, including the median hospital stay duration, leucocyte count, alanine transaminase levels, highest creatinine levels, resuscitated cardiac arrest, and use of norepinephrine, epinephrine, and dopamine.

**Conclusion:**

In an Egyptian cohort, CS incidence was 3.17%, with no mortality difference based on the underlying etiology. Independent predictors of 30-day all-cause mortality included worsening renal function, leucocyte count, resuscitated cardiac arrest, and use of multiple inotropes/vasopressors.

**Graphical abstract:**

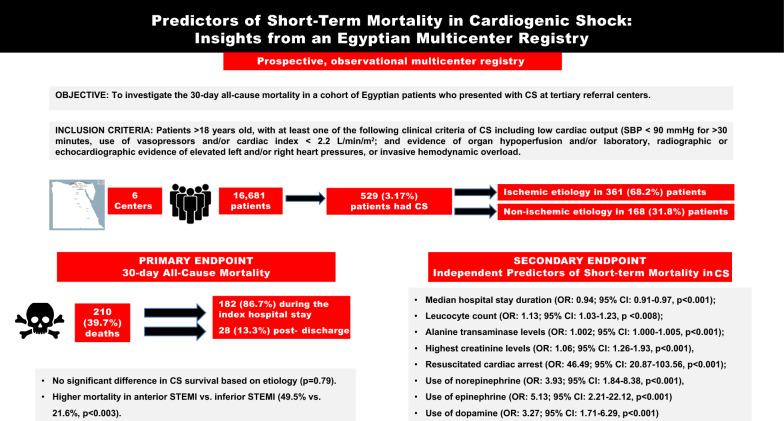

## Background

Cardiogenic shock (CS) is a serious cardiovascular condition, primarily caused by cardiac failure without hypovolemia, leading to low cardiac output and end-organ hypoperfusion. Various definitions have been proposed to encompass the wide range of clinical presentations and outcomes associated with CS [1]. These definitions reflect varying degrees of severity, ranging from mild hemodynamic alterations considered as 'pre-shock' to more severe hemodynamic compromise that can cause multisystem organ failure [2].

Several researchers have investigated the predictors and mortality outcomes of CS to better understand patient characteristics and improve treatment. However, these studies vary significantly in their definitions of CS, patient populations, evaluated predictors, available therapies, and measured outcomes. Most of these studies are observational and prone to selection bias. Furthermore, the data quality can be inconsistent, and the results are often not validated in different populations.

Many studies, such as the SHOCK [2] and IABP II [3] trials, have focused on CS caused by acute coronary syndrome (ACS), neglecting other causes. Primary percutaneous coronary intervention (PPCI) has shown promise in improving outcomes for CS patients with ST-segment elevation myocardial infarction (STEMI), but interventions like intra-aortic balloon pump (IABP), Impella, extracorporeal membrane oxygenation (ECMO), and cardiac assist devices have not shown reduced mortality [4]. The cost and availability of these devices limit their widespread use, especially in developing countries.

This registry aims to provide practical real-life insights into predictors of 30-day all-cause mortality in a cohort of Egyptian CS patients, explore their characteristics, therapeutic interventions, and the underrepresented non-ACS subcategory.

## Methods

### Study population

This prospective multicenter observational registry was conducted between January 2017 and January 2022, and included a total of 16,681 patients admitted to cardiac care units (CCU) across six tertiary medical centers in Egypt. These centers were located in Cairo [New Kasr Al-Ainy Teaching Hospital (28% of patients), Old Kasr Al-Ainy Hospital (24.9%), and Manial Specialized Hospital (8.5%)], Alexandria [Shark Al-Madina Hospital (17.4%) and Sadr Al-Maamoura Hospital (9.1%)], and Behera [Damanhour Hospital (12.1%)]. Among these patients, 529 (3.17%) were diagnosed with CS. The primary outcome was the 30-day all-cause mortality rate. The registry received approval from local ethics committees and followed the guidelines of the Declaration of Helsinki.

Patients aged > 18 years were included if they met at least one of the following criteria: 1) low cardiac output, defined as systolic blood pressure (SBP) < 90 mmHg for over 30 min and/or needing vasopressors to maintain SBP > 90 mmHg and/or cardiac index < 2.2 L/min/m^2^; 2) signs of hypoperfusion, such as urine output < 30 ml/hour, cold/diaphoretic extremities, or altered mental status; and 3) elevation of left and/or right heart pressures, indicated by elevated cardiac biomarkers (e.g., brain natriuretic peptides (BNP) or N-terminal pro-BNP), radiologic volume overload signs on chest X-ray or echocardiography (indicating elevated left ventricular filling pressure), or invasive hemodynamic overload (elevation of mean pulmonary artery pressure or pulmonary capillary wedge pressure) [1–5].

The etiologies of CS were categorized as ischemic or non-ischemic. CS caused by myocardial infarction (MI), including STEMI and non-STEMI (NSTEMI), was classified under ACS etiology. MI followed the Third Universal Definition of MI. STEMI was defined as acute chest pain with ST-segment elevation lasting > 20 min. NSTEMI was defined as acute chest pain without ST-segment elevation. Unstable angina pectoris was identified when there was no rise in cardiac enzymes [4].

Patients excluded from the study were those admitted after being resuscitated from a cardiac arrest, those with terminal cancer, and those with non-cardiogenic shock.

### Data collection

Patients underwent a comprehensive evaluation, including a full history-taking with a special focus on cardiovascular history, clinical presentation, and coexisting medical conditions like chronic kidney disease, pulmonary or neurological disease, and cancer. Risk factors such as hypertension (HTN), diabetes mellitus (DM), smoking, and dyslipidemia were recorded. Treatment, laboratory work-up, electrocardiographic and echocardiographic findings were documented. In the CCU, patients were managed according to local practice and treatment guidelines. The type, duration, sequence, and response to inotropes/vasopressors were recorded, along with anti-thrombotic and anti-hyperlipidemic medications, and gastric protection. Coronary angiography and the modality and timing of revascularization, if performed, were reported.

### End points

The primary endpoint of the study was 30-day all-cause mortality for patients with CS. The secondary endpoints evaluated the predictors of mortality in CS patients with and without ischemic etiology.

### Statistical analysis

The data were analyzed using SPSS (version 21, SPSS Inc., Chicago, Illinois). Continuous data were presented as median (range) due to its abnormal distribution, while categorical data were presented as number (percentage). For quantitative variables, the Mann–Whitney test was used to compare groups, while for qualitative variables, the Fisher's exact test and two-way Chi-squared test were employed. A *p* value of less than 0.05 was considered statistically significant. Survival analysis was conducted using the log-rank test and Kaplan–Meier curves. Additionally, the Cox proportional hazards model was used to test the effects of independent variables on hazards.

## Results

### Clinical and laboratory characteristics

In this study, 529 patients with CS were enrolled from 6 tertiary centers located in different geographic areas. The baseline clinical characteristics of the patients are depicted in Table 1. The mean age of the studied population was 62 ± 14.2 years, with 69.9% of them being men. The most prevalent risk factors observed were DM (56.3%), HTN (42.9%), smoking (32%), and positive family history for coronary artery disease (4.9%). Impaired renal function was the main comorbidity, affecting 23.2%. On admission, the average SBP was 74 ± 8.4 mmHg, diastolic blood pressure was 48 ± 8.7 mmHg, and heart rate was 92 ± 34 beats per minute. The majority of CS cases (68.2%) were of ischemic etiology, while the remaining (31.8%) had a non-ischemic etiology. ACS was identified in 41.6% of all CS patients, with STEMI being the most common presentation (64.5%), followed by NSTEMI in 35.5% of ACS cases. Non-ischemic etiology was found in 31.8% of patients, with decompensated idiopathic dilated cardiomyopathy (12.7%) and valvular heart diseases (9.8%) being the most prevalent conditions. The hospital stay duration for CS patients ranged from 7 to 15 days.

**Table 1:** Baseline demographic characteristics

Variables	All *N* (%) *n* = 529	Survivors *N* (%) *n* = 319	Non-survivors *N* (%) *n* = 210	*p* value
Age, years	62 (53–70)^†^	61 (53–68)^†^	64 (54–73)^†^	**0.01**
Median hospital stay (days)	10 (7–15) †	11 (8–16) †	7 (4–12) †	** < 0.001**
Median ICU stay (days)	9 (6–13) †	10 (7–15) †	7 (4–11) †	** < 0.001**
Women	159 (30.1)	89 (27.9)	70 (33.3)	0.2007
Diabetes Mellitus	298 (56.3)	165 (51.7)	133 (63.3)	**0.009**
Hypertension	227 (42.9)	130 (40.7)	97 (46.2)	0.2432
Smoking				
Current smoker	170 (32.1)	110 (34.5)	60 (28.6)	0.3611
Ex-smoker	75 (14.2)	44 (13.8)	31 (14.8)	
Never smoker	284 (53.7)	165 (51.7)	119 (56.6)	
Renal impairment on presentation	123 (23.2)	71 (22.3)	52 (24.8)	0.528
Worsening of renal function	391 (73.9)	214 (67.1)	177 (84.3)	** < 0.001**
Family history of CAD	26 (4.9)	16 (5)	10 (4.8)	1.0
Prior PCI	125 (23.6)	82 (25.7)	43 (20.5)	0.175
Prior cardiac surgery				0.232
CABG	61 (11.5)	39 (12.2)	22 (10.5)	
Valvular heart surgery	11 (2.1)	4 (1.2)	7 (3.3)	
Congenital heart disease surgery	2 (0.4)	2 (0.6)	0 (0)	
Prior history of cardiac disease				0.232
Ischemic heart disease	181 (34.2)	117 (36.7)	64 (30.5)	
Dilated cardiomyopathy	63 (11.9)	40 (12.6)	23 (10.9)	
Valvular heart disease	44 (8.3)	25 (7.8)	19 (9)	
CTEPH	7 (1.3)	2 (0.6)	5 (2.4)	
Congenital heart disease	6 (1.1)	3 (0.9)	3 (1.4)	
CCM	6 (1.1)	5 (1.6)	1 (0.5)	
Restrictive cardiomyopathy	4 (0.8)	4 (1.2)	0 (0)	
Peripartum cardiomyopathy	2 (0.4)	1 (0.3)	1 (0.50)	
Causes of cardiogenic shock				0.381
Ischemic heart disease	361 (68.2)	217 (68)	144 (68.6)	
ACS	220 (41.6)	126 (39.5)	94 (44.8)	
STEMI	142 (26.8)	82 (25.7)	60 (28.6)	
Anterior STEMI	105 (19.8)	53 (16.6)	52 (24.8)	
Inferior STEMI	37 (7)	29 (9.1)	8 (3.8)	
NSTE-ACS	78 (14.8)	44 (13.8)	34 (16.2)	
Decompensated ICM	141 (26.6)	91 (28.5)	50 (23.8)	
Decompensated DCM	67 (12.7)	43 (13.5)	24 (11.4)	
Decompensated VHD	52 (9.8)	26 (8.1)	26 (12.3)	
Decompensated RSHF	9 (1.7)	4 (1.2)	5 (2.4)	
Myocarditis	9 (1.7)	6 (1.9)	3 (1.4)	
Complete heart block	7 (1.3)	6 (1.9)	1 (0.5)	
Pulmonary embolism	6 (1.1)	5 (1.6)	1 (0.5)	
Decompensated CHD	5 (1.0)	3 (1.0)	2 (1)	
CCM	5 (1.0)	4 (1.2)	1 (0.5)	
Peripartum cardiomyopathy	5 (1.0)	2 (0.6)	3 (1.4)	
RCM	3 (0.5)	3 (1.0)	0 (0)	

Bold indicate *P* value of significance < 0.05

ACS: acute coronary syndrome; CABG: coronary artery bypass graft; CAD: coronary artery disease; STEMI: ST elevation myocardial infarction; ICM: ischemic cardiomyopathy; VHD: valvular heart disease; NSTE-ACS: non-ST elevation acute coronary syndrome; DCM: dilated cardiomyopathy; VHD: valvular heart disease; RSHF: right-sided heart failure; CCM: Chemotherapy-induced cardiomyopathy; CHD: congenital heart disease; CTEPH: chronic thromboembolic pulmonary hypertension; RCM: restrictive cardiomyopathy; †Median, interquartile range

### Incidence of mortality

**Fig. 1:**
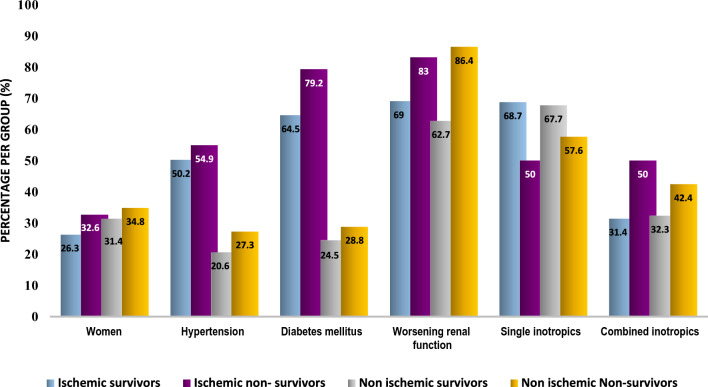
Clinical characteristics of survivors and non-survivors of all cardiogenic shock patients

Within the study, 210 deaths (39.7%) occurred within the initial 30 days of enrollment, with 182 deaths (86.7%) taking place during the index hospital stay, and 28 deaths (13.3%) happening after discharge. Non-survivors of CS patients were older in age, had a higher prevalence of DM, experienced a greater decline in renal functions during hospitalization and had longer hospital stays compared to CS survivors. No significant differences between CS survivors and non-survivors were observed based on the underlying causes of CS. Moreover, CS non-survivors exhibited lower levels of hemoglobin, platelets, and left ventricular ejection fraction, as well as higher total leukocyte counts and worse liver functions tests compared to CS survivors (*p* < 0.001) (Fig. 1 and Table 2).

**Table 2:** Management data for cardiogenic shock patients

Variables	All CS patients *n* = 529	Survivors *n* = 319	Non-survivors *n* = 210	*p* value
Laboratory tests				
Hb (gm/dl)	11.5 (10.1–12.9)^†^	11.9 (10.6–13)^†^	11.05 (9.9–12.4)^†^	** < 0.001**
Plt count (× 1000/mm^3^)	230 (172–297)^†^	240 (180–310)^†^	210 (164.2–271.5)^†^	** < 0.001**
TLC (× 1000/mm^3^)	9.2 (7.6–11.1)^†^	9 (7.3–10.4)^†^	10 (8–12.5)^†^	** < 0.001**
AST (U/L)	70 (38–120)^†^	63 (38–98.5)^†^	94 (48–149)^†^	** < 0.001**
ALT (U/L)	59 (38–111)^†^	52 (34–85.5)^†^	74.5 (44.2–147.7)^†^	** < 0.001**
Urea (mg/dl)	60 (38–95)^†^	51 (37–81.5)^†^	72.5 (44.2–114.2)^†^	** < 0.001**
Baseline creatinine (mg/dl)	1.4 (1.1–2.2)^†^	1.3 (1.1–1.83)^†^	1.75 (1.3–2.6)^†^	** < 0.001**
Highest creatinine (mg/dl)	2.3 (1.5–3.5)^†^	1.9 (1.4–2.7)^†^	3.0 (1.9–3.9)^†^	** < 0.001**
Sodium (mmole/L)	135 (129–139)^†^	135 (130–139)^†^	135 (128–139)^†^	0.689
Potassium (mmole/L)	4.2 (3.9–4.7)^†^	4.2 (3.9–4.6)^†^	4.2 (3.9–4.9)^†^	0.149
HbA1C (gm %)	7.4 (5.8–8.7)^†^	6.9 (5.8–8.5)^†^	7.8 (5.9–8.9)^†^	**0.01**
LVEF (%)	32 (25–38)	34 (28–40)^†^	30 (24–36)	** < 0.001**
Inotropic support				
Single inotrope strategy	324 (61.3)	214 (67.1)	110 (52.4)	** < 0.001**
Combined inotrope strategy	199 (37.6)	99 (31)	100 (47.6)	
First inotrope*				**0.04**
Norepinephrine	185 (35)	113 (35.4)	72 (34.3)	
Dobutamine	62 (11.7)	50 (15.7)	12 (5.7)	
Dopamine	56 (10.6)	38 (11.9)	18 (8.6)	
Epinephrine	21(4)	13 (4.1)	8 (3.8)	
Combined inotrope strategy				
NE + Dopamine	64 (12.1)	29 (9.1)	35 (16.7)	
NE + Dobutamine	62 (11.7)	35 (11)	27 (12.8)	
Dopamine + Dobutamine	39 (7.4)	22 (6.9)	17 (8.1)	0.368
Dopamine + Epinephrine	11 (2.1)	3 (0.9)	8 (3.8)	
Dobutamine + Epinephrine	7 (1.3)	6 (1.9)	1 (0.5)	
NE + Dopamine + Epinephrine	6 (1.1)	0 (0)	6 (2.8)	
NE + Epinephrine	5 (0.9)	0 (0)	5 (2.4)	
NE + Dopamine + Dobutamine	3 (0.6)	2 (0.6)	1 (0.5)	
NE + Dobutamine + Epinephrine	2 (0.4)	2 (0.6)	0 (0)	
Ivabradine	24 (4.5)	14	10	0.84

Bold indicate *P* value of significance < 0.05

ALT: alanine transaminase; AST = aspartate transaminase; Hb: hemoglobin; LVEF: left ventricular ejection fraction; NE: norepinephrine; Plt: platelets; TLC: total leucocytic count; *single inotrope strategy; †Median, interquartile range

The most commonly utilized first inotropic agent was norepinephrine in 185 patients (35%). Dobutamine and dopamine were frequently used as first inotropes in survivors compared to non-survivors. (*p* < 0.04). Among patients who received combination inotropes/vasopressors, the combination of norepinephrine and dopamine was the most frequently used in all CS patients (12.1%). Interestingly, commencing treatment with a single inotropic agent was found to be significantly higher in CS survivors than non-survivors and compared to a combination of vasoactive drugs (Table 2).

### Mortality in CS subgroups according to etiology

In the study, 361 (68.2%) patients were identified with ischemic etiology for CS, while 168 (31.8%) patients had non-ischemic etiology. Interestingly, there was no significant difference between survivors and non-survivors based on the underlying etiology of CS (*p* = 0.79). Among the non-survivors in the ischemic CS group, a higher incidence of DM, worsening renal function defined as an increase ≥ 0.3 mg/dL in the serum creatinine level compared with the baseline value [6, 7], and a usage of initial combined inotropic/vasopressor agents were noted. On the other hand, CS non-survivors with non-ischemic etiology exhibited a higher incidence of worsening renal function, and the primary inotropic agent used was typically noradrenaline (Table 3).

**Table 3:** Cardiogenic shock according to etiology

Variables	All CS patients *n* = 529	Ischemic etiology (*n* = 361)			Non-ischemic etiology (*n* = 168)		
		Survivors (*n* = 217)	Non-survivors (*n* = 144)	*p* value	Survivors (*n* = 102)	Non-survivors (*n* = 66)	*p* value**
Age, years	62 (53–70)^†^	62 (54–69.5)^†^	62 (53–70)^†^	0.3	62 (53–70)^†^	62 (53–70)^†^	0.33
Incidence of mortality		144 (39.9%)			66 (39.3%)		0.79
Women	159 (30.1)	57 (26.3)	47 (32.6)	0.19	32 (31.4)	23 (34.8)	0.64
Diabetes Mellitus	298 (56.3)	140 (64.5)	114 (79.2)	**0.002**	25 (24.5)	19 (28.8)	0.53
Hypertension	227 (42.9)	109 (50.2)	79 (54.9)	0.38	21 (20.6)	18 (27.3)	0.31
Worsening renal function	391 (73.9)	150 (69.1)	120 (83.3)	**0.002**	64 (62.7)	57 (86.4)	**0.0009**
Single inotrope strategy	324 (62)	149 (68.7)	72 (50)	**0.0004**	65 (67.7)	38 (57.6)	0.18
Combined inotrope strategy	199 (38)	68 (31.4)	72 (50)		31 (32.3)	28 (42.4)	
First inotrope*	*n* = 324	*n* = 149	*n* = 72	0.416	*n* = 65	*n* = 38	**0.03**
Norepinephrine	185 (57.1)	78 (52.3)	43 (59.7)		35 (53.8)	29 (76.3)	
Dopamine	56 (17.3)	29 (19.5)	13 (18.1)		9 (13.9)	5 (13.2)	
Epinephrine	21 (6.5)	13 (8.7)	8 (11.1)		0 (0)	0 (0)	
Dobutamine	62 (19.1)	29 (19.5)	8 (11.1)		21 (32.3)	4 (10.5)	

Bold indicate *P* value of significance < 0.05

ACS: acute coronary syndrome; CABG: coronary artery bypass graft; CAD: coronary artery disease; NSTE-ACS: non-ST elevation acute coronary syndrome; PCI: percutaneous coronary intervention; STEMI: ST elevation myocardial infarction; †Median, interquartile range, * single inotrope

### ACS subgroups

Among the patients with ACS, 94 deaths (42.7%) occurred within the first 30 days from enrollment. The mortality rate was not significantly different in patients with STEMI versus those with NSTEMI (42.2% vs. 43.6%, *p* < 0.887). However, the 30-day all-cause mortality was significantly higher in patients with anterior STEMI compared to those with inferior STEMI (49.5% vs. 21.6%, *p* < 0.003).

Both STEMI and non-ST-elevation ACS (NSTE-ACS) non-survivors were older and demonstrated a higher prevalence of DM, worsening renal function, and a higher likelihood of receiving combined inotropic agents as the initial treatment regimen compared to the survivors. Additionally, mortality was significantly higher among those without coronary revascularization (Table 4).

**Table 4:** Cardiogenic shock complicating acute coronary syndrome

Variables	All ACS patients *n* = 220	STEMI patients (*n* = 142)			NSTE-ACS patients (*n* = 78)		
		Survivors (*n* = 82)	Non-survivors (*n* = 60)	*p* value	Survivors (*n* = 44)	Non-survivors (*n* = 34)	*p* value
Age, years	62 (55–69)^†^	60(54.2–68)^†^	62.5(55–71.5)^†^	0.091	63.5 (59–68)^†^	65 (56.2–74.7)^†^	0.696
Women	60 (27.3)	24 (29.3)	18 (30)	0.9251	8 (18.2)	10 (29.4)	0.2461
Diabetes Mellitus	152 (69.1)	51 (62.2)	47 (78.3)	**0.04**	26 (59.1)	28 (82.3)	**0.02**
Hypertension	107 (48.6)	40 (48.8)	37 (62.7)	0.129	18 (40.9)	12 (35.3)	0.6155
Worsening of renal function	157 (71.4)	48 (58.5)	46 (76.7)	**0.02**	32 (72.7)	31 (91.2)	**0.04**
Family history of CAD	17 (7.7)	5 (6.1)	4 (6.7)	0.891	6 (13.6)	2 (5.9)	0.2661
Single inotrope strategy	145 (65.9)	63 (76.8)	33 (55)	**0.006**	33 (75)	16 (47.1)	**0.01**
Combined inotrope strategy	75 (34.1)	19 (23.2)	27 (45)		11 (25)	18 (52.9)	
First inotrope*	*n* = 145	*n* = 63	*n* = 33	0.263	*n* = 33	*n* = 16	
Norepinephrine	90 (62.1)	39 (61.9)	23 (69.8)		19 (57.6)	9 (56.3)	0.482
Dopamine	23 (15.9)	8 (12.7)	5 (15.1)		7 (21.2)	3 (18.7)	
Epinephrine	20 (13.8)	9 (14.3)	5 (15.1)		4 (12.1)	2 (12.5)	
Dobutamine	12 (8.2)	7 (11.1)	0		3 (9.1)	2 (12.5)	
Revascularization done	149 (67.7)	74	45	**0.01**	35	5	** < 0.001**
Type of revascularization	*n* = 159	*n* = 74	*n* = 45		*n* = 35	*n* = 5	
PCI	121 (76.1)	63 (85.1)	33 (73.4)	**0.01**	24 (68.6)	1 (20)	**0.03**
PTCA	2 (1.3)	0 (0)	2 (4.4)		0 (0)	0 (0)	
CABG	18 (11.3)	0 (0)	3 (6.7)		11 (31.4)	4 (80)	
Thrombolysis	10 (6.3)	5 (6.8)	5 (11.1)		0 (0)	0 (0)	
Pharmacoinvasive	8 (5)	6 (8.1)	2 (4.4)		0 (0)	0 (0)	

Bold indicate *P* value of significance < 0.05

ACS: acute coronary syndrome; STEMI: ST elevation myocardial infarction; NSTE-ACS: non-ST elevation myocardial infarction; PCI: percutaneous coronary intervention; PTCA: percutaneous transluminal coronary angioplasty; CABG: coronary artery bypass graft

Multivessel disease (MVD), defined as the presence of one or more significant coronary stenoses in addition to the infarct-related artery, was found in 67.1% of ACS patients, and this group had a significantly higher mortality rate compared to those without MVD (*p* < 0.001 for STEMI patients). Percutaneous coronary intervention (PCI) was associated with better survival compared to coronary artery bypass grafting in both STEMI and NSTE-ACS patients (Table 4).

Furthermore, a strategy of culprit vessel-only PCI with no further intervention was associated with a higher mortality compared to culprit PCI followed by total revascularization during the index hospitalization (Table 5). The left anterior descending artery was the most common culprit vessel in both STEMI and NSTE-ACS patients, accounting for 57.4% and 70%, respectively. The left main artery as a culprit was associated with a significantly higher 30-day all-cause mortality (*p* < 0.001). A significant association was found between successful revascularization and 30-day survival rate (*p* < 0.01 for STEMI, and < 0.001 for NSTE-ACS).

**Table 5:** Cardiogenic shock complicating acute coronary syndrome

Variables	All ACS patients	STEMI patients			NSTE-ACS patients		
		Survivors	Non-survivors	*p* value	Survivors	Non-survivors	*p* value
Culprit vessel	*n* = 155	*n* = 70	*n* = 45		*n* = 35	*n* = 5	
Left main artery	23 (14.8)	6 (8.6)	16 (35.6)		1 (2.8)	0	0.09
LAD	94(60.7)	42 (60)	24 (53.3)	**0.001**	25 (71.5)	3 (60)	
LCX	11 (7.1)	5 (7.1)	2 (4.4)		2 (5.7)	2 (40)	
RCA	27 (17.4)	17 (24.3)	3 (6.7)		7 (20)	0	
Failed PCI	6 (9.8)	6 (26.1)			0 (0)		
Renal impairment	31(50.9)	2 (8.7)			29 (76.4)		
Death before CA	5 (8.2)	0 (0)			5 (13.1)		
Patient refusal	3 (4.9)	2 (8.7)			1 (2.6)		
Non-PCI capable hospital	3 (4.9)	0 (0)			3 (7.9)		
Extent of CAD	*n* = 155	*n* = 70	*n* = 45	**0.001**	*n* = 35	*n* = 5	0.057
1 vessel	51 (32.9)	32 (45.7)	15 (33.3)		4 (11.4)	0 (0)	
2 vessels	40 (25.8)	20 (28.6)	4 (8.9)		16 (45.7)	0 (0)	
3 vessels	64 (41.3)	18 (25.7)	26 (57.8)		15 (42.9)	5 (100)	
Revascularization in patients with more than one vessel CAD							
Culprit-only revascularization		19 (61.3)	20 (86.9)	**0.03**			
Total revascularization during index hospitalization		12 (38.7)	3 (13.1)				

Bold indicate *P* value of significance < 0.05

ACS: acute coronary syndrome; STEMI: ST elevation myocardial infarction; NSTE-ACS: non-ST elevation myocardial infarction; LAD: left anterior descending artery; RCA: right coronary artery; PCI: percutaneous coronary intervention; CA: coronary angiography; CAD: coronary artery disease

### Predictors of 30-day all-cause mortality in CS patients

To identify the most relevant variables that predicted mortality in patients with CS, a multivariate regression analysis was completed. The analysis revealed several independent predictors of mortality. These included the median hospital stay duration (OR 0.94; 95% CI 0.91–0.97, *p* < 0.001), total leucocytic count (OR 1.13; 95% CI 1.03–1.23, *p* < 0.008), alanine transaminase (ALT) levels (OR 1.002; 95% CI 1.000–1.005, *p* < 0.001), highest creatinine levels (OR 1.06; 95% CI 1.26–1.93, *p* < 0.001), occurrence of resuscitated cardiac arrest (OR 46.49; 95% CI 20.87–103.56, *p* < 0.001), norepinephrine use (OR 3.93; 95% CI 1.84–8.38, *p* < 0.001), epinephrine use (OR 5.13; 95% CI 2.21–22.12, *p* < 0.001), and dopamine use (OR 3.27; 95% CI 1.71–6.29, *p* < 0.001).

## Discussion

To our knowledge, this multicenter registry represents the largest prospective study on CS in Egypt to date. The study included 529 patients, with 361 of them being diagnosed with ischemic etiology. These patients were recruited from 6 different centers in three cities. In contrast to the majority of previous studies that primarily focused on patients diagnosed with CS secondary to acute MI (AMI), our study aimed to address all CS phenotypes and etiologies including the often-neglected population of CS patients without an ischemic trigger. This particular group of patients has been overlooked or underrepresented in prior research, making our study unique in its approach and contribution to the field.

Due to the lack of a consensus on a universal definition and the utilization of different criteria by various studies [5], our registry employed a simple and practical CS definition. This definition allowed for clinical fulfillment without the need for extensive expertise, enabling the rapid recognition and inclusion of a larger number of patients. Unlike many previous definitions [5], our approach involved considering clinical symptoms and signs, as well as noninvasive methods such as chest X-ray and echocardiography, to diagnose low cardiac output and overload patterns. This approach aimed to avoid the need for invasive hemodynamic assessment.

### Mortality rates in patients with cardiogenic shock

Historically, the mortality rate for CS was around 70% between the mid-1970s and late 1980s. However, with the introduction of early revascularization concepts in ischemic patients in the early 2000s, the mortality rate dropped to approximately 40%. Furthermore, in the early 2010s, the mortality rate further decreased to around 30–40% [3, 8].

In our study, we observed a high 30-day mortality rate of 39.7% with 86.7% taking place during the index hospital stay, and 13.3% happening after discharge. Mortality was significantly higher among older individuals and those with DM, regardless of gender or the presence of HTN or ischemic or non-ischemic etiology.

Previous studies showed that in-hospital mortality rates of CS following MI vary considerably across different medical centers [9–16]. A study involving 351 patients with CS following AMI reported a higher in-hospital mortality rate of 44.7%, which may be related to participants having a slightly higher mean age (65.41 ± 7.78 years vs. 62 ± 14.2 years) compared to our study. Similar to our findings, age and DM were significant factors associated with mortality, while gender showed no significant difference. However, HTN also played a significant role in predicting mortality, unlike our study where it showed no difference [16].

In the CSWG (Cardiogenic Shock Working Group) registry version 1 (V1) and external cohorts, three distinct CS phenotypes were identified: I, "non-congested," II, "cardiorenal," and III, "cardiometabolic" shock. These phenotypes were retrospectively identified in 796 patients in version 2 (V2) of the registry. The in-hospital mortality rates for phenotypes I, II, and III were 23%, 41%, and 52%, respectively. Interestingly, the use of mechanical circulatory support was found to be associated with significantly increased mortality in the cardiorenal phenotype, but not in the non-congested or cardiometabolic phenotypes. These findings suggest that the identification of specific CS phenotypes may be valuable in designing future clinical trials and developing tailored management algorithms for each phenotype [17].

In contrast to our study, a retrospective study involving patients presenting with AMI-CS at a tertiary care center reported a 30-day mortality rate of 46%. The median age of the patients in this study was slightly higher at 63 years. Similar to our study, higher baseline creatinine levels were significantly associated with mortality. However, in contrast to our study, advanced age and DM were not predictors of 30-day mortality in this retrospective study [18].

### Predictors of mortality in patients with cardiogenic shock

In line with our registry, advanced age was associated with significantly increased mortality in previous studies [19–22]. However, this finding was not reproducible in a subgroup analysis of patients with ACS, as was reported in the Melbourne Interventional group registry. The Melbourne registry showed no difference in 1-year outcomes in AMI patients with CS, who were older than 75 years compared to younger patients [23].

Additionally, there have been conflicting findings regarding the relationship between gender and mortality in patients with cardiogenic shock [9]. In our study, there was no difference in 30-day mortality between males and females, although the majority of patients presenting with CS were males.

DM also yielded inconsistent results, with some studies indicating adverse outcomes in diabetics, while others showed no such association [19, 21, 24]. In line with our findings, a meta-analysis of 15 observational studies revealed that diabetic patients had a higher risk of in-hospital mortality compared to those without DM. The increased risk of mortality was also observed at 30 days and 1 year post-discharge [25].

Acute renal failure has been identified as a predictor of mortality, indicating the severity of shock [8, 26]. In our registry, deteriorating kidney function and higher initial levels of serum creatinine were both associated with higher mortality rates. A retrospective study conducted at a single-center examined the outcomes of patients with CS who were on Impella-CP and experienced acute kidney injury (AKI). The study found a significant association between AKI and 30-day mortality [27]. The CardShock study demonstrated that AKI occurred in more than one-third of the patients with CS and that AKI determined by creatinine levels was strongly correlated with an increased risk of 90-day mortality [1, 28]. The findings from these two studies align with our registry data, further supporting the association between AKI and mortality in CS patients.

### Causes of cardiogenic shock

The most significant causes of CS in our study were ACS, decompensated ischemic cardiomyopathy, and decompensated non-ischemic cardiomyopathy. Similarly, in the cardiogenic shock prognosis (CSP) score study [29], as well as the Cardshock study [1], ACS was perceived as the most common etiology of CS.

While our registry found no difference in 30-day all-cause mortality based on the cause of CS, other studies have reported conflicting results. An observational study, which included 978 patients, reported that non-ischemic CS accounted for 52% of all cases (vs. 31.8% of cases in our study), and these patients were more likely to present in a worse clinical condition and had a significantly higher risk of 30-day in-hospital mortality [30]. On the other hand, the CardShock study found that non-ACS causes accounted for 19% of cases and were associated with a better prognosis compared to ACS causes [1]. The discrepancies in findings between these studies could be attributed to differences in study populations, design and treatment strategies.

In our study, there was no significant difference in mortality between STEMI and NSTE-ACS. Similarly, the GUSTO-IIb trial, which included 12 084 patients, did not find any significant difference in 30-day mortality between CS patients presenting with STEMI and those with non-ST elevation (63.0% vs. 72.5%, respectively) [31].

### Management strategies

#### Medications

Inotropic and vasoactive drugs may improve hemodynamics and organ dysfunction. However, data comparing inotropic agents in CS are scarce and non-randomized. In the SOAP II trial, there was no difference between dopamine and norepinephrine in adverse outcomes and mortality in septic shock. However, in the subgroup of CS patients, dopamine was associated with a greater number of adverse events, but there was no significant difference in mortality [32]. In the optima CC trial, epinephrine was associated with a higher incidence of refractory shock compared to norepinephrine [33]. On the other hand, there was no evidence to support the use of a certain inotrope over another in a systematic review [34].

In our registry, there was a significant difference in 30-day all-cause mortality between CS patients according to the first inotrope used. This difference was mainly driven by higher mortality with the use of norepinephrine as the first inotrope compared to lower mortality with the use of dobutamine. Additionally, the use of a combination of inotropic agents as a first-line strategy was associated with a higher 30-day mortality compared to a single agent. However, the possibility of selection bias with the use combination therapy in more severe cases, leading to worse outcomes, cannot be excluded.

In the present study, a small group of 24 patients received ivabradine, and it did not affect the mortality rates. However, the small number of patients limits the importance of this finding. Another small, but randomized, study evaluated heart rate lowering with ivabradine in 58 patients with CS complicating AMI and showed the safety of using ivabradine in those patients [35]. However, larger trials are required to further investigate the efficacy and safety of ivabradine.

#### Revascularization strategies

In concordance with our registry results, the SHOCK trial demonstrated that early revascularization led to improved survival in patients with cardiogenic shock (CS) complicating acute myocardial infarction (AMI). However, while our study indicated a survival advantage as early as 30 days, the benefits observed in the SHOCK trial were evident at 6 months [2].

Most STEMI patients, in our study, underwent a culprit-only PCI strategy with or without total revascularization during the index hospitalization, and patients who had staged total revascularization during the same index hospitalization had significantly lower 30-day mortality. Similarly, the CULPRIT-SHOCK trial, which included 706 patients with AMI, demonstrated a significant reduction in the primary outcome, consisting of 30-day mortality or renal replacement therapy, with a strategy of culprit lesion–only PCI (with the option of staged total revascularization during hospitalization) compared to immediate multivessel PCI [36].

### Limitations

The current study has some limitations. Firstly, it is an observational study and not a randomized trial. However, despite this, the data from this real-world study provides invaluable information on the management strategies of CS in our country and reports, for the first time, on its outcomes. Secondly, the noninvasive diagnostic methods may not have provided a comprehensive assessment of the hemodynamic status and severity of CS. Without invasive methods, there is a potential for misclassifying patients who may have had other causes of shock, such as septic or hypovolemic shock, as CS, which may have led to inappropriate management and suboptimal outcomes in some cases. Furthermore, most centers in our region do not use mechanical circulatory support. Surprisingly, our outcomes were as good as centers that used advanced technology and sophisticated life support measures. Lastly, being an observational study, treatment details were left to the physician's discretion at each hospital, although treatments like coronary intervention were based on the most recent guidelines.

## Conclusions

This multicenter registry is the largest prospective study on CS in Egypt, offering valuable information on the management strategies employed and presenting comprehensive outcome data. It investigates all CS phenotypes and etiologies, including the frequently overlooked population of CS patients without an ischemic trigger. In this observational study, the 30-day mortality rate for CS with different underlying etiologies was 39.7%. Several factors were predominantly associated with mortality, including older age, worsening of renal function, DM, failure of revascularization in ACS patients, and the presence of a left main culprit artery in STEMI patients. From all clinical and therapeutic variables, worsening renal function, total leucocytic count, resuscitated cardiac arrest, and use of multiple inotropes/vasopressors were identified as independent predictors of 30-day all-cause mortality in CS patients.

## Data Availability

The dataset supporting the results and conclusions of this article will be available from the corresponding author on request.

## Abbreviations

ACS: Acute coronary syndrome
AKI: Acute kidney injury
ALT: Alanine transaminase
AMI: Acute myocardial infarction
BNP: Brain natriuretic peptide
CCU: Cardiac care unit
CS: Cardiogenic shock
DM: Diabetes mellitus
ECMO: Extracorporeal membrane oxygenation
HTN: Hypertension
IABP: Intra-aortic balloon pump
MI: Myocardial infarction
MVD: Multivessel disease
NSTE-ACS: Non-ST-elevation acute coronary syndrome
NSTEMI: Non-ST-segment elevation myocardial infarction
STEMI: ST-segment elevation myocardial infarction
SBP: Systolic blood pressure
